# Self-Efficacy and Self-Care as Risk Factors for Ischemic Stroke: Development and Validation of a Nomogram

**DOI:** 10.3390/jcm12175665

**Published:** 2023-08-31

**Authors:** Al Rasyid, Uke Pemila, Siti Aisah, Salim Harris, Elvan Wiyarta, Marc Fisher

**Affiliations:** 1 Department of Neurology, Faculty of Medicine, Universitas Indonesia, Dr. Cipto Mangunkusumo National Hospital, Jakarta 10430, Indonesia; 2 Directorate of Health Service Governance, Indonesian Ministry of Health, Jakarta 12940, Indonesia; 3 Department of Medical Surgery, Faculty of Nursing, Universitas Indonesia, Dr. Cipto Mangunkusumo National Hospital, Jakarta 10430, Indonesia; 4 Department of Medical Science, Faculty of Medicine, Universitas Indonesia, Dr. Cipto Mangunkusumo National Hospital, Jakarta 10430, Indonesia; 5 Beth Israel Deaconess Medical Center, Boston, MA 02215, USA

**Keywords:** ischemic stroke, patient education, prevention, Indonesia, self-confidence

## Abstract

Background: This study addresses the knowledge gap on how self-efficacy and self-care affect stroke risk as factors and develops a valuable tool for clinicians to assess stroke risk. Methods: From January 2022 to January 2023, this nested-case control study was conducted. Medical data including gender, age, ethnicity, locality, education, marital status, employment, caregiver, social environment, blood viscosity, Barthel Index, modified Rankin Scale (mRS), stroke risk score, self-care score, and self-efficacy score were collected. Logistic regression was used to predict stroke risk, and a nomogram was developed and validated. Results: 240 patients were included in the analysis. Stroke risk score (OR: 3.513; *p* = 0.005), self-efficacy score (OR: 0.753; *p* = 0.048), and self-care score (OR: 0.817; *p* = 0.018) were predictors of ischemic stroke. Internal validation was carried out, with a C-index of 0.774, and the Hosmer–Lemeshow test indicated a good fit (*p* = 0.92). The calibration plot also shows that this nomogram model has good calibration abilities. The decision curve analysis (DCA) results show a threshold probability range of 10–95%. Conclusion: A nomogram has been developed with good validity, calibration, and clinical utility, including self-care and self-efficacy as risk factors for predicting ischemic stroke.

## 1. Introduction

Ischemic stroke is a cerebrovascular disease characterized by an obstructed blood vessel supplying oxygen to the brain [1]. It accounts for about 87% of all strokes in developed countries and is the most common form of stroke [2]. Globally, it is an important cause of disability and mortality, with a significant clinical and economic burden [3]. The World Health Organization (WHO) ranks stroke as the second main cause of mortality and the leading cause of disability worldwide [3]. Despite advances in stroke treatment and management, the incidence and prevalence of stroke continue to rise globally [4].

Several risk factors have been identified for ischemic stroke, including hypertension, diabetes, smoking, and atrial fibrillation [5]. These risk factors are often associated with lifestyle choices, diet, and exercise [5]. Conversely, recent research has centered on the function of self-efficacy and self-care in relation to ischemic stroke [6,7]. Self-efficacy refers to a person’s belief in their capacity to perform a specific behavior or activity [8]. Self-care encompasses a variety of activities individuals engage in to maintain their health and well-being [9]. These factors have been shown to significantly influence the incidence and treatment of chronic diseases, such as stroke [7].

Despite the potential significance of self-efficacy and self-care in stroke prevention, little is known about their effect on the risk of ischemic stroke. Most studies on stroke risk have focused on conventional risk factors, such as hypertension and diabetes, but have not included self-efficacy and self-care measures [10]. This knowledge gap emphasizes the need for a greater understanding of the impact of these variables on the risk of stroke and the development of tools that can effectively incorporate these factors into risk prediction models.

Several obstacles might explain why self-efficacy and self-care are not included in stroke risk prediction models. Identifying appropriate measures for these factors is one of the most significant obstacles. Self-efficacy and self-care are difficult to measure objectively due to their complexity [11]. Self-report measures, such as questionnaires, are frequently employed to evaluate these constructs but are frequently susceptible to biases, such as social desirability and response bias [12]. Moreover, self-report measures may not adequately capture the actual effect of self-efficacy and self-care on stroke risk [12].

Incorporating self-efficacy and self-care into stroke risk prediction models also presents the challenge of selecting relevant clinical and demographic variables to include in the model. Traditional risk factors for stroke, such as hypertension and diabetes, are well-established and have been confirmed by many studies [5]. However, the inclusion of self-efficacy and self-care in risk prediction models requires the identification of additional variables that can effectively capture the impact of these factors on the risk of stroke. Identifying these variables necessitates an in-depth comprehension of the interplay between self-efficacy, self-care, and traditional stroke risk factors.

Despite these obstacles, the construction and validation of a nomogram that integrates self-efficacy and self-care as risk factors for ischemic stroke have the potential to yield important insights for stroke prevention and management. A nomogram is a graphical representation of a predictive model that estimates the probability that an event will occur given a set of predictors [13]. Nomograms have been utilized in various disciplines, including medicine, to forecast outcomes and guide decision-making [13].

This study aims to address the knowledge gaps regarding the influence of self-efficacy and self-care on stroke risk and to develop a practical tool for clinicians and researchers to assess stroke risk. We hypothesize that self-efficacy and self-care are risk factors that significantly influence the incidence of stroke, apart from conventional factors. Ultimately, this study has the potential to shed light on the role of self-efficacy and self-care in stroke prevention and contribute to the development of more accurate risk prediction instruments for stroke.

## 2. Methods

### 2.1. Study Design and Eligibility Criteria

This nested-case control study was conducted at the Cipto Mangunkusumo National Hospital, Jakarta, Indonesia, from January 2022 to January 2023. In January 2022, the Faculty of Medicine Ethics Committee at Universitas Indonesia accepted the research protocol, given the protocol number KET-4/UN2.F1/ETIK/PPM.00.02/2022. A total of 240 patients were retrospectively enrolled from medical records.

Patients who met the eligibility criteria were included in the study. The inclusion criteria for this study were patients diagnosed with mild to moderate ischemic stroke (National Institutes of Health Stroke Scale (NIHSS) ranging from 1 to 15) with confirmed brain imaging examinations, aged more than 18 years, able to speak Indonesian, and with a stable medical condition for at least 48 h post-stroke. The exclusion criteria of this study were patients with incomplete medical record data, patients with chronic neurological conditions other than stroke, end-stage cancer, pre-stroke dementia, severe cognitive impairment, and psychiatric impairment. Patients who met the inclusion and exclusion criteria were enrolled for analysis in the study according to the World Medical Association’s Code of Ethics (Declaration of Helsinki) [14].

### 2.2. Sample Calculation and Data Collection

The samples were selected from as many eligible samples from the medical data as possible. The samples selected in this study are samples in another ongoing cohort study. Therefore, this research design is called nested-case control. At the start of the cohort study, all patients had similar characteristics. All patient data was taken at this early stage. Progress of the patient to stroke or not was followed afterward. The minimum sample size was determined using a confidence level of 95%, or 5% alpha, and a power of 80%, or 20% beta. This investigation requires a minimum sample size of 90 participants per group.

Various demographic and clinical characteristic data were obtained from the patient’s medical records, which include gender, age, ethnicity, locality, education, marital status, employment, caregiver, social environment, blood viscosity, Barthel Index, modified Rankin Scale (mRS), stroke risk score, self-care score, and self-efficacy score. To reduce bias, all data on stroke patients and non-stroke patients were collected by two independent evaluators. The research team collected data on a spreadsheet, verified it, and then sent it to an independent statistician. Functional outcome was measured by mRS score [15] and Barthel index [16], while blood viscosity (expressed in centipoise) was measured by Digital Microcapillary Instrument [17].

The stroke risk score was assessed using the Feigin Stroke Risk Score [5]. The Feigin Stroke Risk Score has been validated in Indonesia and is extensively used [18]. This stroke risk score comprises the following factors: age, blood pressure, blood sugar, cholesterol, BMI, family history of stroke, alcohol consumption, cigarette smoking, physical activity, and nutrition. Depending on the patient’s risk factors, each component (excluding diet) is assigned a value between 0 and 3, with 3 being the highest. The diet component is evaluated using a 0 or 1 number, with 0 indicating that the patient follows the recommendations and 1 indicating that they do not. The Feigin Stroke Risk Score, which ranges from 0 to 28, is determined by aggregating the numbers associated with each component. A score of 0 indicates a minimal risk for stroke, whereas a score of 28 indicates an extremely high risk for stroke.

Self-care and self-efficacy scores were assessed using the Hypertension Self-Care Instrument [19], validated in Indonesia [18]. The instrument has seventeen components. The patient responds on a scale from 1 to 4 for each item. In assessing self-care, 1 means “never”, 2 means “rarely”, 3 means “often”, and 4 means “always”. In assessing self-efficacy, a value of 1 corresponds to “not sure”, a value of 2 to “not sure enough”, a value of 3 to “sure”, and a value of 4 to “very sure”. Both the self-care and self-efficacy components have the same questions, only the responses are different. All components of these questions were assessed in each patient by trained health workers. The cumulative score for self-care and self-efficacy on the Hypertension Self-Care Instrument is 68. The greater the patient’s self-care and self-efficacy, the greater the Hypertension Self-Care Instrument’s value.

### 2.3. Statistical Analysis

Before analysis, Microsoft Excel was used to input data gathering into a main table (Microsoft Corp, Redmond, WA, USA). The statistical analyses were performed using R 4.1.0 (R Core Team, Vienna, Austria). For univariate analysis, all categorical variables are expressed as frequencies and analyzed using a chi-square test. All numerical variables are expressed as means (standard deviation) and analyzed using simple logistic regression. Missing data were excluded. A *p*-value less than 0.05 is considered statistically significant. After that, multivariate analysis using multiple logistic regression was also performed by including predictor variables with *p*-values less than 0.20. Using the “rms” component of the R programming language, the resulting variables from the multivariate analysis were used to construct the nomogram. An internal validation procedure used one thousand bootstraps resamples to reduce the overfitting bias. Using the “DynNom” component of the R—4.3.1 software, a web application was created to facilitate the use of nomograms [20].

The C-index ranged from 0.5 to 1 to evaluate the nomogram’s discriminatory potential. The higher the C-index, the more discriminative the model. Consequently, a value of 0.5 indicated no discrimination, 0.7–0.8 indicated acceptable discrimination, 0.8–0.9 indicated excellent discrimination, and larger than 0.9 indicated exceptional discrimination [21]. A calibration curve and the Hosmer–Lemeshow test were used to evaluate the calibration capability of the nomogram. The calibration curve illustrates the similarity between predicted and actual outcomes [21]. A Hosmer–Lemeshow test with a *p*-value = 0.92 indicated that the nomogram model was well-fitting [21].

## 3. Results

This study analyzed 240 patients, 120 (50%) ischemic stroke patients and 120 (50%) non-stroke patients from January 2022 to October 2022. Overall, there were 118 males and 122 females with a mean age of 56.42 ± 12.79 years in the study. These patients’ demographic data and clinical characteristics are presented in Table 1.

Univariate analysis was performed to compare various parameters of ischemic stroke events. The analysis results show that six parameters have a significant relationship with the incidence of stroke. These parameters are blood viscosity (odds ratio [OR]: 1.42; 95% confidence interval [CI]: 1.22–1.65; *p* < 0.001), Barthel Index (OR: 0.98; 95% CI: 0.97–0.99; *p* < 0.001), Modified Rankin Scale (OR: 1.24; 95% CI: 1.03–1.51; *p* = 0.026), stroke risk score (OR: 3.52; 95% CI: 2.39–5.18; *p* < 0.001), self-care score (OR: 0.70; 95% CI: 0.64–0.77; *p* < 0.001), and self-efficacy score (OR: 0.68; 95% CI: 0.60-0.76; *p* < 0.001), as shown in Table 1.

Multivariate analysis of parameters with a *p*-value below 0.2 shows a significant relationship to the incidence of stroke (Table 2). Stroke risk score (OR: 3.513; *p* = 0.005), self-efficacy score (OR: 0.753; *p* = 0.048), and self-care score (OR: 0.817; *p* = 0.018) were predictors of ischemic stroke. The multivariate analysis becomes the basis of the nomogram development for calculating the risk of stroke.

**Figure 1:**
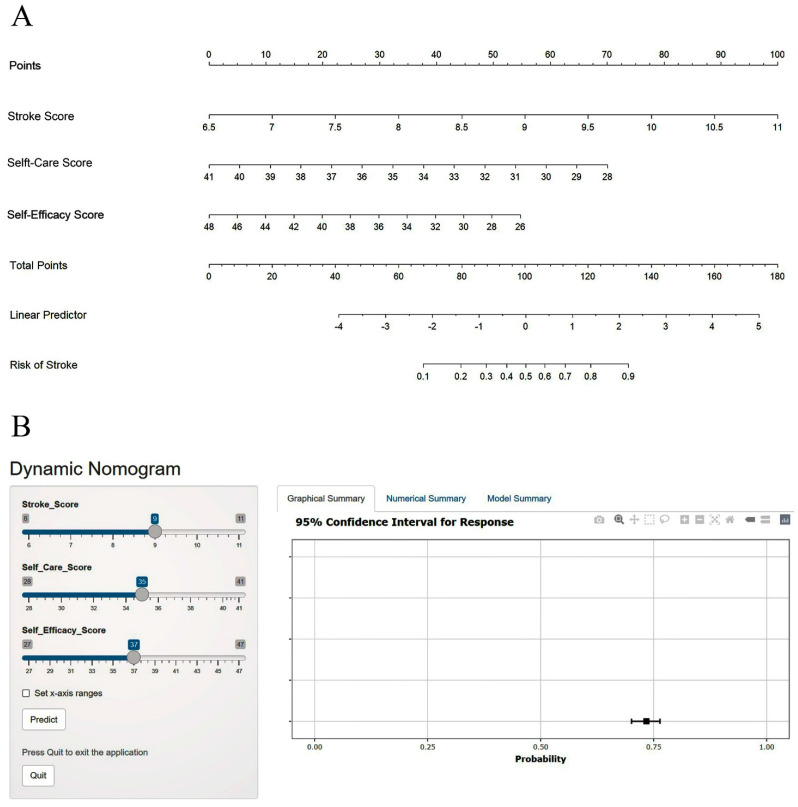
Nomogram to predict the risk of stroke. (**A**) Nomogram predicting the risk of stroke based on stroke score, self-care score, and self-efficacy score. (**B**) An example of a web screen display of the risk of stroke prediction model application.

Through the analysis of the regression model, a nomogram was constructed using three predictors, as shown in Figure 1A. Furthermore, to be more applicable and easily used by clinicians and patients, a web version of the nomogram (Figure 1B) has been developed and can be accessed at https://elvanwiyarta.shinyapps.io/StrokeNomogram/ accessed on 15 March 2023. The calculated risk of stroke can be easily determined by using the nomogram or by inputting the values of the variables in the web application above. For example, a patient with a stroke risk score of 9 (55 points), a self-care score of 35 (32 points), and a self-efficacy score of 37 (28 points) will have a total score of 115. If plotted on the nomogram (Figure 1A), this patient’s stroke risk is about 70%. This value can be calculated more accurately through a dynamic nomogram (Figure 1B), which gives the patient’s stroke risk of 73.4% (95% CI: 71.4–75.3%).

**Figure 2:**
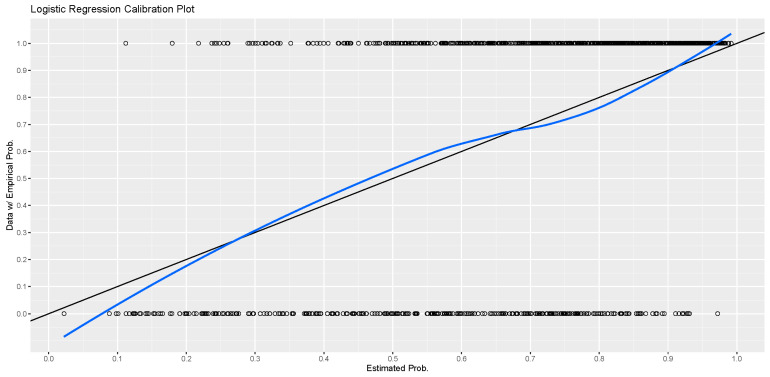
Nomogram calibration graph for estimating stroke risk (blue line). The y-axis represents the actual probability of stroke, while the x-axis represents the predicted probability of stroke. The reference line is at 45 degrees (black line), which denotes perfect calibration.

Internal validation was carried out to assess the validity of this nomogram using the bootstrap technique with 1000 resamples. The validity test results showed excellent discrimination ability for assessing the risk of stroke with a C-index of 0.774. The Hosmer-Lemeshow test also indicated a good fit of the prediction nomogram (*p* = 0.92). In addition, the calibration plot also shows that this nomogram model has good calibration abilities, as seen in Figure 2.

**Figure 3:**
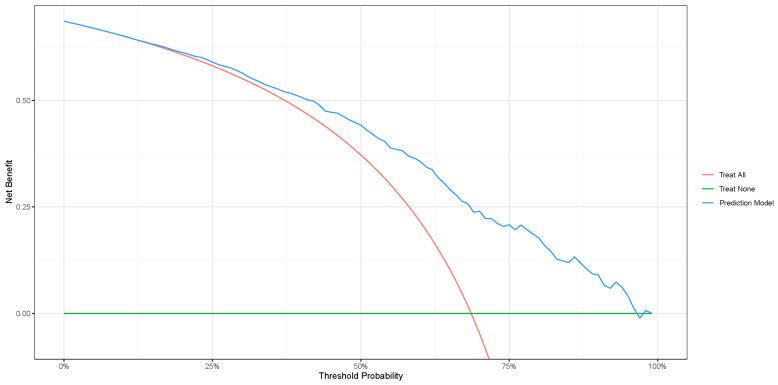
Decision curve for the nomogram. The probability level is shown on the x-axis, and the net benefit is shown on the y-axis.

A decision curve analysis (DCA) was carried out to assess the clinical utility of this nomogram, which can be seen in Figure 3. The DCA results show that this nomogram’s threshold probability range might be more beneficial than the “treat-all” or “treat-none” strategy, with a threshold probability range of 10–95%. For example, based on a 50% risk of stroke, the nomogram added a net benefit of 7% compared with the “treat-all” or “treat-none” strategy.

## 4. Discussion

In this study, self-efficacy and self-care are factors that predict the risk of stroke. In addition to using the Feigin Stroke Risk Score as a conventional predictive model, adding the self-efficacy score and self-care score to the nomogram significantly increases the model’s discriminatory ability and clinical utility. The Feigin Stroke Risk Score is a predictive model commonly used to predict stroke risk. In contrast, self-efficacy and self-care are often not considered in determining a patient’s stroke risk factors.

Based on the results of this study, the lower the patient’s self-efficacy score (OR: 0.753; *p* = 0.048) and self-care score (OR: 0.817; *p* = 0.018), the higher the stroke risk, and vice versa. Self-efficacy and self-care are two significant psychological factors identified as predictors of stroke risk. It has been demonstrated that these factors influence individuals’ behavior and capacity to manage their health and lifestyle, thereby affecting the likelihood of developing a stroke.

Self-efficacy can be defined as an individual’s confidence in their ability to effectively complete a specific task or accomplish a particular objective [8]. This belief is influenced by various factors, including past experiences, social support, and personal qualities like motivation, resilience, and optimism [8]. Self-efficacy is a significant predictor of health outcomes because it affects an individual’s ability to engage in health-promoting behaviors, such as regular exercise, healthy diet, and medication adherence [8].

In contrast, self-care refers to individuals’ actions to maintain and improve their physical and mental health [9]. Self-care encompasses a variety of practices, such as exercise, healthy nutrition, stress management, medication adherence, and routine health examinations. Self-care is a crucial predictor of health outcomes because it has been shown to reduce the risk of developing chronic diseases like stroke and enhance overall health and well-being [9].

Self-efficacy and self-care were identified as predictors of the risk of stroke in this study, in addition to conventional predictors such as hypertension, diabetes, diet, or exercise. This result is because these psychological factors influence an individual’s behavior and capacity to manage their health effectively.

For example, a study found that higher levels of self-efficacy were associated with a lower risk of stroke in individuals with hypertension [22]. The study found that individuals with high levels of self-efficacy were more likely to engage in health-promoting behaviors, such as regular exercise and healthy eating, which reduced their risk of developing a stroke [22]. Similarly, another study found that self-care practices, such as medication adherence and regular health check-ups, were associated with a lower risk of stroke in individuals with diabetes [23].

These findings highlight the importance of considering psychological factors such as self-efficacy and self-care in predicting the risk of stroke. While conventional predictors such as hypertension and diabetes are important risk factors for stroke, they do not capture the full range of factors influencing an individual’s health and behavior. By considering psychological factors such as self-efficacy and self-care, healthcare professionals can better understand an individual’s health and well-being and tailor interventions to address specific needs and behaviors.

It is important to note that self-efficacy and self-care are not mutually exclusive from conventional predictors of stroke risk, such as hypertension, diabetes, diet, or exercise, because these factors will likely be interconnected and influence each other [24]. For example, an individual with hypertension may have lower levels of self-efficacy due to the stress and anxiety associated with managing their condition. This may lead to a lower likelihood of engaging in health-promoting behaviors such as regular exercise and healthy eating, further increasing their risk of stroke [24].

All of the predictors described are the basis for making the nomogram. The nomogram developed in this study has good validity and calibration values. In addition, this nomogram also has good clinical utility with a range of 10–95%. The development of this nomogram is intended to facilitate the implementation of a stroke risk prediction model based on the factors discussed above. With the existence of a conventional and dynamic nomogram, it is hoped that it will make it easier for clinicians and patients to assess risk factors.

In the DCA, we are aware of negative results in this model, which theoretically means that using this model actually causes disadvantages to stroke patients compared to not using it. However, this value is only a notch, and again rises above the treat none line. This can occur allegedly due to the variability of the data taken. Therefore, further research can be conducted to validate our research externally.

There are several limitations to this study. First, because this study is retrospective, not a prospective study, there will be some biases related to predictor factor analysis, which might undermine the implications of the model being developed. Second, nomogram validation was only done internally because no testing dataset exists. Further research is needed to validate this nomogram with external data from various research sites.

## 5. Conclusions

Self-care and self-efficacy are risk factors for stroke prediction, apart from conventional risk factors. This study developed a nomogram with good validity, calibration, and clinical utility, including self-care and self-efficacy assessments for predicting ischemic stroke patients.

## Figures and Tables

**Table 1:** Baseline Demographic and Clinical Characteristics of Study Participants.

Parameter	Stroke		*p*-Value	OR	95% CI	
	Yes (*n* = 120)	No (*n* = 120)			Min	Max
Gender						
Male	63 (53.4%)	55 (46.6%)	0.37	1.31	0.78	2.17
Female	57 (46.7%)	65 (53.3%)				
Age	57.43 (13.6)	55.4 (11.9)	0.22	1.01	0.99	1.03
Ethnicity						
Javanese	52 (46.8%)	59 (53.2%)	0.83	1 (ref)		
Sundanese	39 (52.0%)	36 (48.0%)		1.23	0.68	2.21
Bataknese	11 (55.0%)	9 (45.0%)		1.39	0.53	3.61
Others	18 (52.9%)	16 (47.1%)		1.28	0.59	2.76
Locality						
Urban	65 (49.6%)	66 (50.4%)	1.00	0.97	0.58	1.61
Rural	55 (50.5%)	54 (49.5%)				
Education						
Senior high school or above	89 (49.4%)	91 (50.6%)	0.88	0.92	0.51	1.64
Junior high school or below	31 (51.7%)	29 (48.3%)				
Marital Status						
Married	111 (51.2%)	106 (48.8%)	0.49	1.51	0.62	3.69
Unmarried	9 (40.9%)	13 (59.1%)				
Employment						
Employed	38 (45.8%)	45 (54.2%)	0.42	0.77	0.45	1.32
Unemployed	82 (52.2%)	75 (47.8%0				
Caregiver						
Parent	8 (61.5%)	5 (38.5%)	0.69	1 (ref)		
Siblings	15 (50.0%)	15 (50.0%)		0.66	0.17	2.36
Spouse	23 (44.2%)	29 (55.8%)		0.49	0.14	1.72
Children	74 (51.0%)	71 (49.0%)		0.65	0.20	2.09
Social Environment						
Family	79 (47.0%)	89 (53.0%)	0.31	1 (ref)		
Friends	25 (54.3%)	21 (45.7%)		1.34	0.69	2.58
Neighbors	16 (61.5%)	10 (38.5%)		1.80	0.77	4.20
Blood Viscosity (cP)	6.48 (1.51)	5.30 (2.11)	<0.001 *	1.42	1.22	1.65
Barthel Index	51.96 (27.21)	66.44 (27.13)	<0.001 *	0.98	0.97	0.99
mRS	2.62 (1.42)	2.23 (1.26)	0.026 *	1.24	1.03	1.51
Stroke Risk Score	14.72 (2.95)	6.81 (2.24)	<0.001 *	3.52	2.39	5.18
Self-Care Score	22.39 (5.37)	39.95 (6.08)	<0.001 *	0.70	0.64	0.77
Self-Efficacy Score	21.73 (4.70)	45.04 (7.39)	<0.001 *	0.68	0.60	0.76

* *p*-value less than 0.05 is considered statistically significant. Univariate analysis was performed using the Chi-square test and simple logistic regression. Data are presented as means (SD) or numbers (%). CI: confidence interval; cP: centi Poise; mRS: modified Rankin Scale; OR: odd ratio; Ref: reference; SD: standard deviation.

**Table 2:** Multivariate analysis of stroke risk factor.

Predictor	β	SE β	Wald	df	*p*-Value	e^β^ (Odds Ratio)
Stroke Risk Score	1.257	0.445	7.959	1	0.005 *	3.513
Self-Care Score	−0.284	0.144	3.911	1	0.048 *	0.753
Self-Efficacy Score	−0.202	0.086	5.553	1	0.018 *	0.817
Constant	7.131	4.938	2.085	1	0.149	

* *p*-value less than 0.05 is considered statistically significant. SE: standard error; df: degree of freedom; e: Euler number ≈ 2.718.

## Data Availability

This published paper and its Supplementary Material include all data produced or analyzed during the research.

## Supplementary Materials

The following supporting information can be downloaded at: https://www.mdpi.com/article/10.3390/jcm12175665/s1.
