# Supplementary material for: Self-Efficacy and Self-Care as Risk Factors for Ischemic Stroke: Development and Validation of a Nomogram
Source: J Clin Med. 2023 Aug 31;12(17):5665. doi: 10.3390/jcm12175665 (PMC10489054; doi:10.3390/jcm12175665)
Supplement: Supplementary file 1 [file jcm-12-05665-s001.zip › jcm-2511063-SI.pdf]

S1. R code for the nomogram development and validation

```
library(rms)

n<- 1000

set.seed(1000)

d<-data.frame(Stroke_Score = rnorm(n,9,0.6),

            Self_Care_Score = rnorm(n,35,2),

            Self_Efficacy_Score = rnorm(n,37,3))

L<-7.131 + 1.257*d$Stroke_Score + -0.284*d$Self_Care_Score + -0.202*d$Self_Efficacy_Score

p<-plogis(L)

d$y<- rbinom(n=1000, size = 1, prob = p)

table(d$y)

ddist <- datadist(d)

f<- lrm(y ~ Stroke_Score + Self_Care_Score + Self_Efficacy_Score, data = d)

f


# Create nomogram

options(datadist = 'ddist')

nom <- nomogram(f, fun = plogis, funlabel = "Risk of Stroke")

plot(nom)


#new data for calibration

new_d <- data.frame(Stroke_Score = rnorm(1000,9,0.6),

                  Self_Care_Score = rnorm(1000,35,2),

                  Self_Efficacy_Score = rnorm(1000,37,3))

new_d$y<- rbinom(n=1000, size = 1, prob = p)



#calibration

library(predtools)

library(magrittr)

library(dplyr)
```

```
library(ggplot2)

f<- glm(y ~ Stroke_Score + Self_Care_Score + Self_Efficacy_Score,d, family=binomial)

pd <- with(d, data.frame(y = ifelse(y, 1, 0),
                        prob = predict(f, type = "response")))
ggplot(pd, aes(prob, y)) +
  geom_point(shape = 21, size = 2) +
  geom_abline(slope = 1, intercept = 0) +
  geom_smooth(method = stats::loess, se = FALSE) +
  scale_x_continuous(breaks = seq(0, 1, 0.1)) +
  scale_y_continuous(breaks = seq(0, 1, 0.1)) +
  xlab("Estimated Prob.") +
  ylab("Data w/ Empirical Prob.") +
  ggtitle("Logistic Regression Calibration Plot")

#Decision curve analysis
library(dcurves)
library(gtsummary); library(dplyr); library(tidyr)
library(labelled)

mod2 <- glm(y ~ Stroke_Score + Self_Care_Score + Self_Efficacy_Score, d, family = binomial)
tbl_regression(mod2, exponentiate = TRUE)

d<- d %>%
  mutate(
    strokepred =
      broom::augment(mod2, type.predict = "response") %>%
      pull(".fitted")
  )
```

```
dca(y ~ strokepred,
    data = d,
    thresholds = seq(0, 1, 0.01),
    label = list(strokepred = "Prediction Model")
) %>%
  plot(smooth = FALSE)

#cds table
dca(y ~ strokepred,
    data = d,
    as_probability = "strokepred",
    thresholds = seq(0, 1, 0.05)
) %>%
  as_tibble() %>%
  select(label, threshold, net_benefit) %>%
  gt::gt() %>%
  gt::fmt_percent(columns = threshold, decimals = 0) %>%
  gt::fmt(columns = net_benefit, fns = function(x) style_sigfig(x, digits = 3)) %>%
  gt::cols_label(
    label = "Strategy",
    threshold = "Decision Threshold",
    net_benefit = "Net Benefit"
  ) %>%
  gt::cols_align("left", columns = label)


#dynamic nomogram
library(DynNom)

f<- glm(y ~ Stroke_Score + Self_Care_Score + Self_Efficacy_Score,d, family=binomial)
```

```
DynNom(f)

DNbuilder(f, d, clevel = 0.774)
```
